# Reading habits contribute to the effects of display direction on product choice

**DOI:** 10.1371/journal.pone.0209837

**Published:** 2018-12-26

**Authors:** Atsunori Ariga

**Affiliations:** Hiroshima University, Kagamiyama, Higashi-hiroshima, Hiroshima, Japan; The University of Queensland, AUSTRALIA

## Abstract

It has been shown that people process assortment variety more efficiently in horizontal displays than in vertical displays, owing to human visual characteristics that favor the horizontal direction. Consequently, the number and variety of products chosen tends to increase when they are arranged horizontally. I show that this horizontal display advantage can be modulated by culture, especially writing and reading habits. When Japanese participants, who write and read text vertically as well as horizontally, chose products in horizontal and vertical displays, horizontal displays did not consistently increase the variety of products chosen. In other words, the horizontal display advantage was eliminated (Experiments 1A and 1B). However, when Japanese readers initially read horizontally, it led to a robust advantage for the horizontal display. Similarly, initial vertical reading resulted in a vertical display advantage (Experiments 2 and 3). These results suggest that horizontal displays are not always advantageous, and that optimal display direction for product choice is affected by reading habits.

## Introduction

Although excessive variety in product assortments can make people less likely to consume [1], the efficient perception of assortment variety can attract more consumers and increase purchase behavior [2,3]. For example, when consumers efficiently categorize products via structural designs or labels, the variety and quantity of products purchased increases, as does their satisfaction with their choices [4,5]. Grouping products using color cues guides consumers’ visual processing and behavior [6]. That is, the perception of variety affects consumer choice.

Spatially allocating products in a display is a simple and useful technique for facilitating consumer perception of assortment variety. A recent study demonstrated that the direction of the display (horizontal versus vertical product arrangement) affects consumer choice. Deng and colleagues [7] hypothesized that horizontal displays have an advantage over vertical displays for visual processing, because the human visual field and the direction of dominant eye movements are weighted in favor of the horizontal direction. They measured the variety of products purchased from horizontally or vertically arranged assortments in a shopping mall. Customers who visited the horizontal display purchased a greater variety of products than those who visited the vertical display, resulting in a greater quantity of products purchased. The authors interpreted this *horizontal display advantage* as a result of human visual characteristics oriented for efficient perception in the horizontal plane.

The horizontal display advantage is robust: it has been observed with various types of stimuli not only in the field but also in controlled laboratory experiments. For example, in Deng and colleagues’ Study 3, the advantage was consistently elicited when ten images depicting a chocolate assortment were displayed on a screen, regardless of whether they were presented for short (3 s) or long (15 s) durations. Nevertheless, it would be premature to conclude that the horizontal display advantage is universal. Thus, it is necessary to identify factors that modulate or negate any advantage in terms of practical realization. With respect to this issue, a number of psychological studies on visual cognition have suggested that visual processing strategy depends strongly on culture [8–13]; I consider, therefore, that the horizontal display advantage in variety choice [7] may be susceptible to cultural differences, which is the focus of the present paper.

For example, people from Western cultures (“Westerners”) write and read text from left to right, thus horizontally. Accordingly, they represent numbers on a mental number line oriented from left to right, in which smaller numbers are located leftward and larger numbers are located rightward [14]. Based on this directional representation, when a small number is presented at the center of a screen, Westerners automatically shift their covert attention to the left side of the screen and efficiently process visual information in that region [15]. Conversely, visual attention covertly shifts to the right side when a large number is presented. Interestingly, the small-left and large-right association is reversed for people from Arabic cultures (“Arabians”) because they write and read text from right to left, and they establish a right-to-left mental number line [12]. Arabians more quickly report the side of the larger number in a pair when it is presented on the left side of a screen, following their right-to-left writing and reading habits [16]. In short, converging evidence in the field of visual cognition demonstrates that visual attention is covertly controlled in response to directional reading and writing habits [8,12]. If efficient visual processing of the product assortment in horizontal displays explains the advantageous effects observed by Deng and colleagues [7], it is plausible that the horizontal display advantage can be modulated by cultural practices such as reading habits.

In the present study, I hypothesize that the advantages of display direction in terms of product choice are susceptible to culture, in particular on the practices of horizontal or vertical reading. Although Deng and colleagues [7] ruled out any effect of reading history when analyzing eye movements, this reflected only “overt” attentional processing [17,18]. As described above, reading culture modulates not only overt but also “covert” attention [8,12,15]; the latter functions independently of eye movement. Thus, it is possible that directional reading habits may modulate, or indeed override, the horizontal display advantage that basically reflects horizontally favored visual characteristics. Here, I predict that, for Japanese readers, who write and read text not only horizontally (from left to right) but also vertically (from top to bottom), no specific display direction is advantageous in terms of product choice (as revealed by the absence of horizontal display advantages in Experiments 1A and 1B). I further investigate whether display direction effects can be modulated by priming participants with horizontal or vertical text (thus, both vertical and horizontal display advantages were explored in Experiments 2 and 3).

My goal was to determine whether any independent variable exerted any effect. As traditional null-hypothesis significance tests (NHSTs) do not gather evidence in favor of the null hypothesis [19,20], I used default Bayesian tests [21] in this study. Bayes factors were treated as measures of evidence for or against effects of interest. Briefly, a Bayes factor indicates the ratio of the likelihood that the data obtained favor a statistical model including the effects of interest to the likelihood that they favor a model that excludes those effects. Rather than *p*-values, I report *B*_*10*_ values of Bayes factors and use terminology denoting the magnitudes of such effects, as introduced by previous work [22,23]. A *B*_*10*_ value greater than 1 affords evidence of a statistical effect, whereas a *B*_*10*_ value less than 1 evidences the null hypothesis. Furthermore, Bayesian tests allow experimenters to stop sampling optionally [24], thus guarding against so-called “*p-hacking*” or ad-hoc corrections to the NHSTs. In this study, I ceased sampling when the *B*_*10*_ value for the effect of interest attained 6 or 1/6 (i.e., affording moderate evidence as defined by Wagenmakers and colleagues [23]).

## Experiment 1A

First, I investigated the existence of the horizontal display advantage reported by Deng and colleagues [7] for Japanese participants. Based on my hypothesis, I predicted a null effect of the horizontal display on product choice.

### Method

All experiments were reviewed and approved by the Institutional Review Board of Hiroshima University, Japan. I obtained written informed consent from all participants both before and after the experiment.

I recruited 40 Japanese participants (17 males, mean age 19.80 years, SD = 1.00 years) from the student community. All participants reported normal or corrected-to-normal visual acuity. Participants were blinded to the purpose of the study.

**Fig 1:**
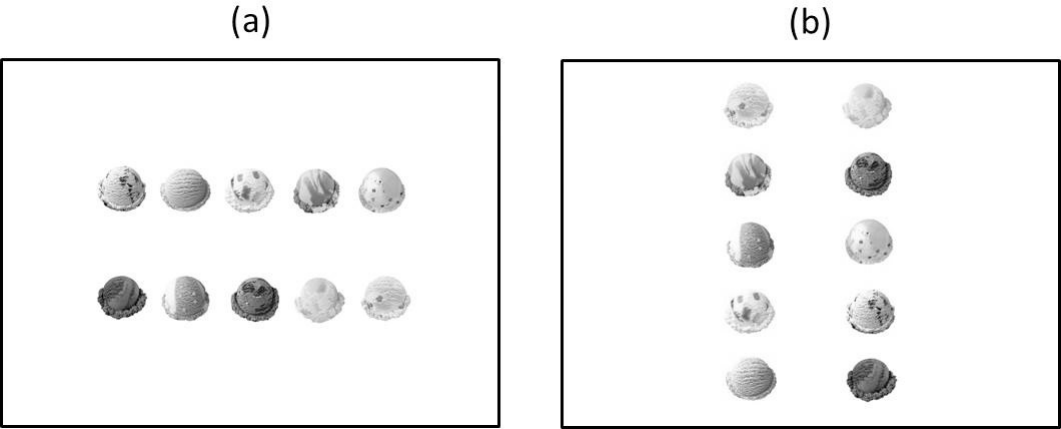
Schematic illustration of (a) the horizontal and (b) vertical displays used in Experiment 1A.

The stimuli and procedure were similar to those used in Deng and colleagues [7], Study 3. Each horizontal (Fig 1A) and vertical (Fig 1B) display consisted of ten images depicting different flavors of ice cream, which were full-color and randomly selected from forty flavors of ice cream, against a white background. Each image subtended 7.1°× 7.1° in visual angle. In a horizontal display, five stimuli were aligned horizontally and separated by 2.8° between the neighboring two images in each upper and lower row. The upper and lower rows were separated by 7.8° above and below the center of the screen. In a vertical display, five stimuli were aligned vertically and separated by 2.8° between neighboring images in each left and right row. The left and right rows were separated by 7.8° left and right of the center of the screen. They were presented on a 30-inch screen. Participants viewed the screen from a distance of 16 inches.

After participants pressed the keyboard space key, a fixation point appeared at the center of the screen for 1 s. Then, a horizontal or vertical display was presented for a duration of 2 s or 15 s; these durations were determined in a pilot study to provide sufficient (or insufficient) time for participants to recognize all varieties in the assortment. The task was to answer a question after viewing each display: “Consider choosing seven ice creams from this assortment. You can choose either seven of the same flavor, or up to seven different flavors. How many different flavors would you like to choose?” (1 to 7). This question measured the variety of unique flavors chosen and thus served as an index of variety choice, as in Deng and colleagues [7].

The participants were subjected to two display directions (horizontal and vertical) × 2 durations (2 s and 15 s) × 1 trial, in a total of 4 trials, the order of which was randomized between participants. The same flavor did not appear repeatedly for any participant.

### Results and discussion

**Fig 2:**
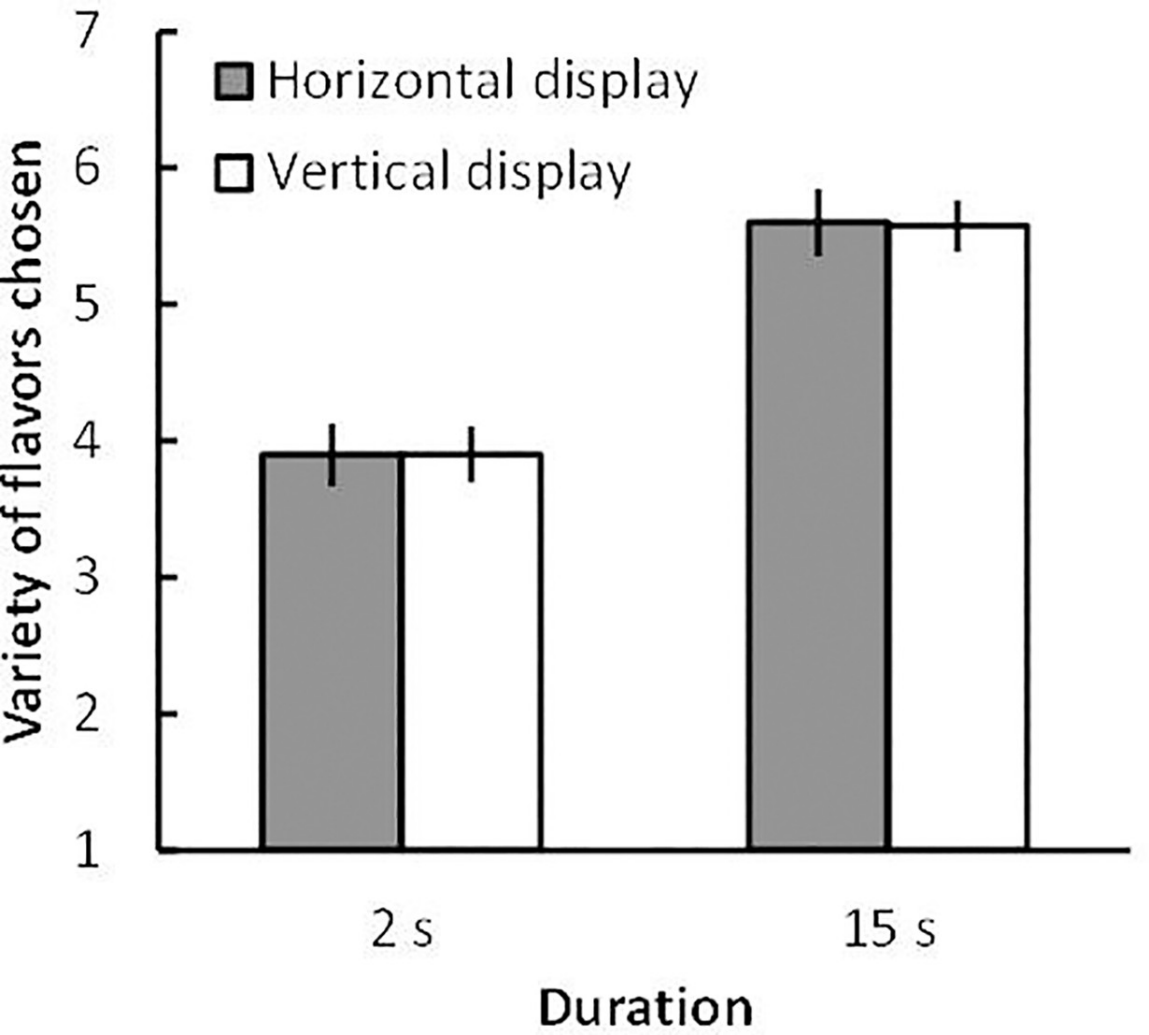
Mean numbers of flavors chosen (variety choices) under each condition of Experiment 1A. Error bars indicate standard error of the mean.

The variety of flavors that participants chose (variety choice) was averaged across participants for each condition. A within-subject 2 (display direction: horizontal and vertical) × 2 (duration: 2 s and 15 s) Bayesian analysis of variance (ANOVA) was conducted. The data provided moderate evidence that display direction favored the null (Fig 2) (*B*_*10*_ = 1/6.02, *partial η*^*2*^ = 0.00), but extreme evidence of an effect of duration (*B*_*10*_ = 1.39 × 10^14^, *partial η*^*2*^ = 0.60). The data afforded moderate evidence that the interaction between these factors favored the null (*B*_*10*_ = 1/4.20, *partial η*^*2*^ = 0.00).

As predicted, the horizontal display afforded no advantage, unlike previous reports to the effect that variety choice increased when the displays were horizontal [7]. This is probably attributable to the fact that Japanese participants usually write and read texts both vertically and horizontally. However, it remained possible that Japanese participants did not perceive display directions in the same manner as Westerners. Given the Japanese reading culture, participants might perceive only the vertical alignment of each two-item row in the horizontal display, thus eliminating the horizontal display advantage. To exclude this possibility, I distorted the vertical alignment of the horizontal display and the horizontal alignment of the vertical display in Experiment 1B to allow participants to perceive only a single direction within each display based on the Gestalt law of good continuation. Furthermore, in Experiment 1B, I measured the number of flavors identified as well as the variety of flavors chosen to check the validity of duration (thus, whether 2 s was too short and 15 s sufficiently long for participants to process the assortment).

## Experiment 1B

### Method

I recruited 42 Japanese participants (19 males, mean age = 20.17 years, SD = 1.23 years) from the student community. All participants reported normal or corrected-to-normal visual acuity. All were blinded to the purpose of the study.

**Fig 3:**
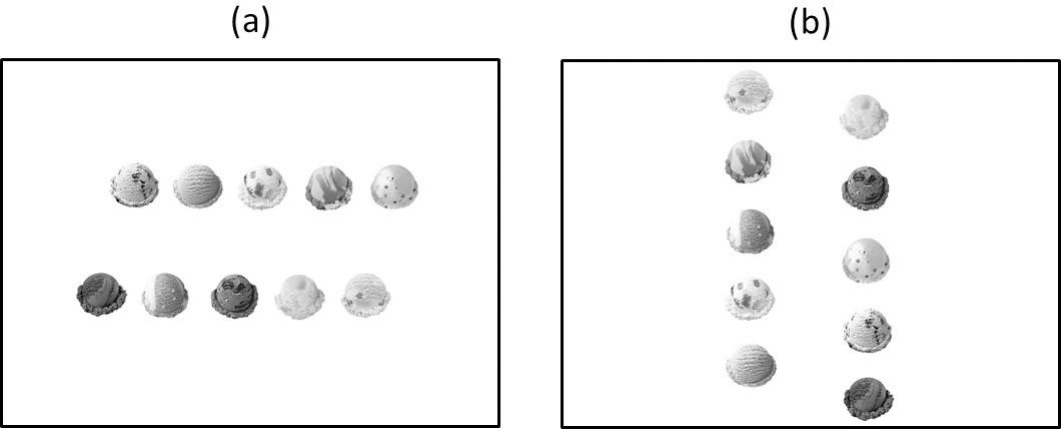
Schematic illustration of (a) the horizontal and (b) vertical displays used in Experiments 1B, 2 and 3.

With the exception of the following changes, the stimuli, apparatus, and procedure were the same as those of Experiment 1A. In the horizontal display, the upper and lower rows did not vertically align; the upper row was located 2.8° right of the center of the screen and the lower row 2.8° left of the center (Fig 3A). In the vertical display, the left and right rows did not horizontally align; the left row was located 2.8° above the center of the screen and the right row 2.8° below the center (Fig 3B). The participants answered two questions after viewing each display. First, the number of flavors identified in the assortment was directly measured, to confirm that the 2-s duration was too short and the 15-s duration sufficiently long to process the stimuli. I asked: “How many flavors did you identify in this assortment?” (answers: 1 to 10). Second (and importantly), the variety of unique flavors chosen was measured, as in Experiment 1A. The participants were subjected to two display directions (horizontal and vertical) × two durations (2 s and 15 s) × one trial, to form a total of four trials.

### Results and discussion

**Fig 4:**
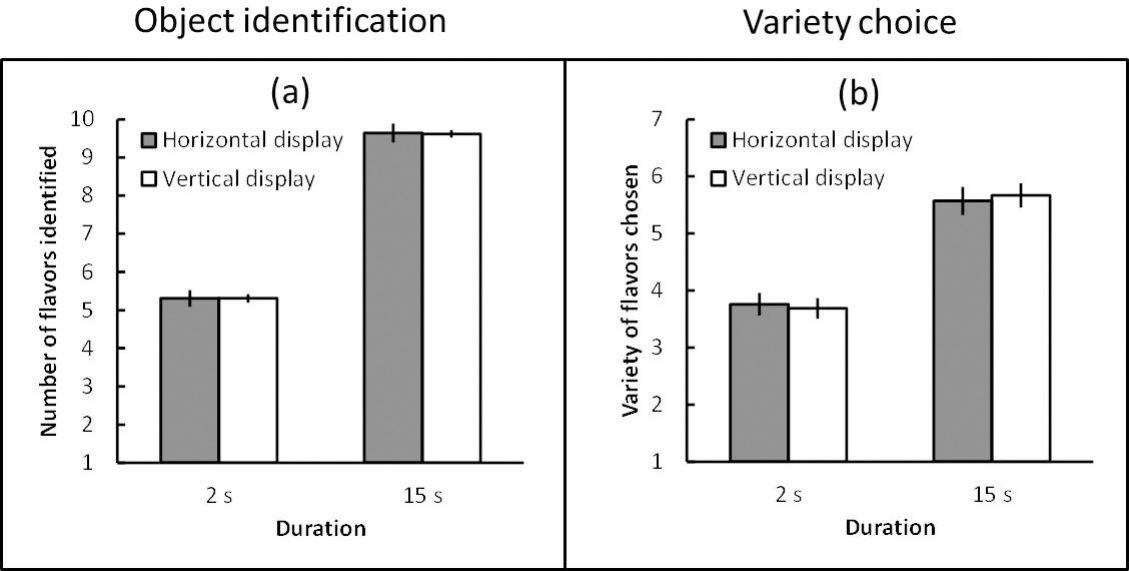
(a) Mean number of flavors identified (object identification) under each condition and (b) mean variety of flavors chosen (variety choice) under each condition in Experiment 1B. Error bars indicate standard error of the mean.

The number of flavors identified (object identification) and the variety of flavors chosen (variety choice) were averaged across the participants under each condition. A within-subject 2 (display direction: horizontal and vertical) × 2 (duration: 2 s and 15 s) Bayesian ANOVA was performed in terms of object identification and variety choice. The object identification data provided moderate evidence that the display direction favored the null (Fig 4A) (*B*_*10*_ = 1/5.29, *partial η*^*2*^ = 0.00), but extreme evidence of an effect of duration (*B*_*10*_ = 6.27 × 10^57^, *partial η*^*2*^ = 0.92). The data afforded moderate evidence that the interaction between these factors favored the null (*B*_*10*_ = 1/4.61, *partial η*^*2*^ = 0.00). The variety choice data indicated moderate evidence that the display direction favored the null (Fig 4B) (*B*_*10*_ = 1/6.02, *partial η*^*2*^ = 0.00), but extreme evidence of an effect of duration (*B*_*10*_ = 1.43 × 10^20^, *partial η*^*2*^ = 0.72). The data provided moderate evidence that the interaction between these factors favored the null (*B*_*10*_ = 1/4.24, *partial η*^*2*^ = 0.01).

First, Japanese participants identified more flavors and chose a greater variety of flavors in the 15 s trial than in the 2 s trial. These results confirmed that the 15 s display duration was sufficiently long for participants to identify almost all varieties in the assortment, while the 2 s duration was too short, which affected the variety of flavors chosen. Second, I found, again, that the horizontal display afforded no advantage in terms of variety choice, even though the participants were likely to perceive a single direction in each display.

In contrast to previous studies, the horizontal display advantage was apparently absent in Japanese participants. Therefore, the results of Experiments 1A and 1B support my hypothesis that the advantageous direction of the assortment display in terms of variety choice is susceptible to cultural differences in reading habits, or on whether participants are familiar with horizontal or vertical reading.

## Experiment 2

To provide further evidence for my hypothesis, I investigated whether vertical or horizontal reading selectively primes participants, activating the corresponding direction of visual information processing and thus modulating the advantage of display direction for Japanese participants. Based on my hypothesis, I predicted the effects of both horizontal and vertical displays on product choice.

### Method

I recruited 42 Japanese participants (22 males, mean age = 20.26 years, SD = 1.07 years) from the student community. All participants reported normal or corrected-to-normal visual acuity. Participants were blinded to the purpose of the study.

**Fig 5:**
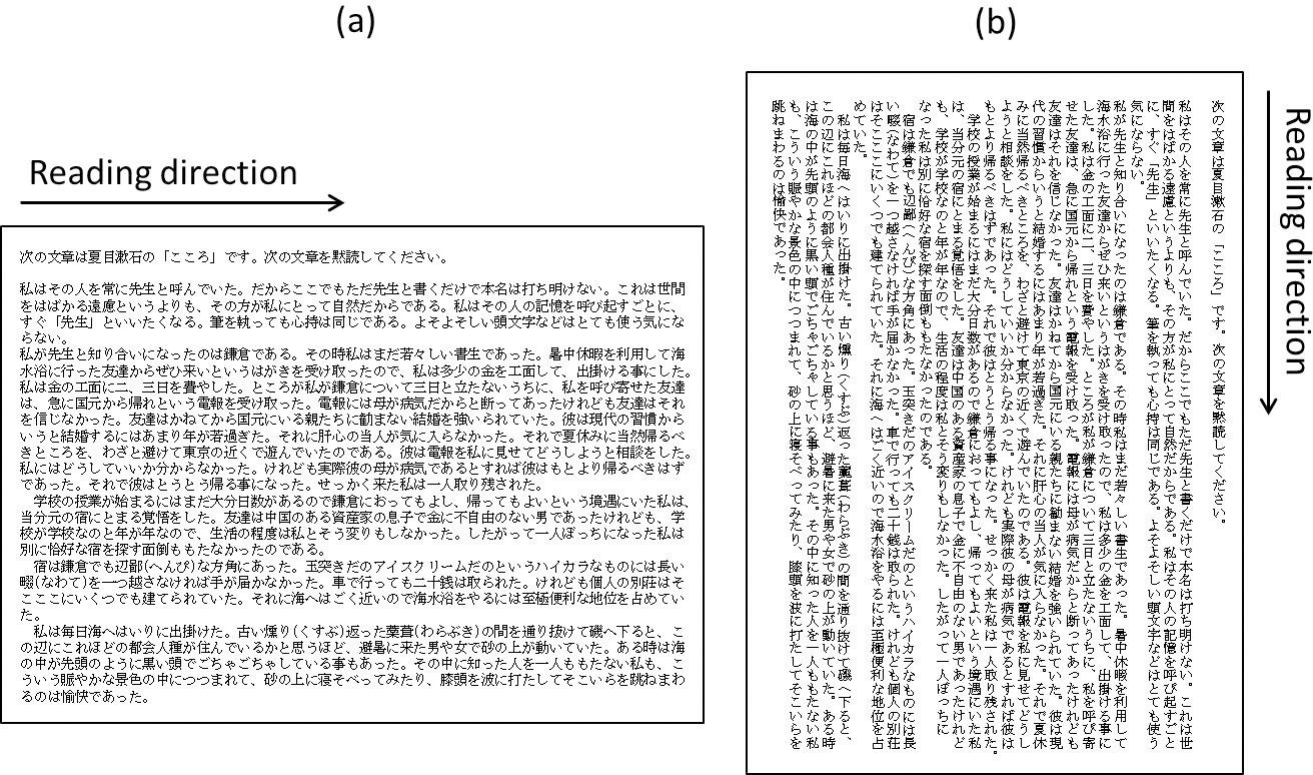
(a) Stimulus texts printed horizontally on horizontally oriented A4 paper under the horizontal-reading condition and (b) printed vertically on vertically oriented A4 paper under the vertical-reading condition in Experiments 2 and 3. (The term “Reading direction” and the arrow were not present in the experiment itself).

The stimuli, apparatus, and procedure were the same as those used in Experiment 1B, except that the experiment consisted of prime (reading) and probe (choice) sessions. To activate the direction of visual processing, participants were initially required to read, silently, several passages (1,103 characters) of a well-known Japanese novel, “*Kokoro*” [25]. The novel was printed in horizontal writing on horizontally oriented A4 paper (the horizontal-reading condition, Fig 5A) or in vertical writing on vertically oriented A4 paper (the vertical-reading condition, Fig 5B) with a font size of 10.5. After the reading task, participants performed the choice task as described in Experiment 1B. Twenty-two participants were assigned to the horizontal-reading condition, and the other 20 to the vertical-reading condition. They were exposed to 2 display directions (horizontal and vertical) × 2 durations (2 s and 15 s) × 1 trial, for a total of 4 trials.

### Results and discussion

**Fig 6:**
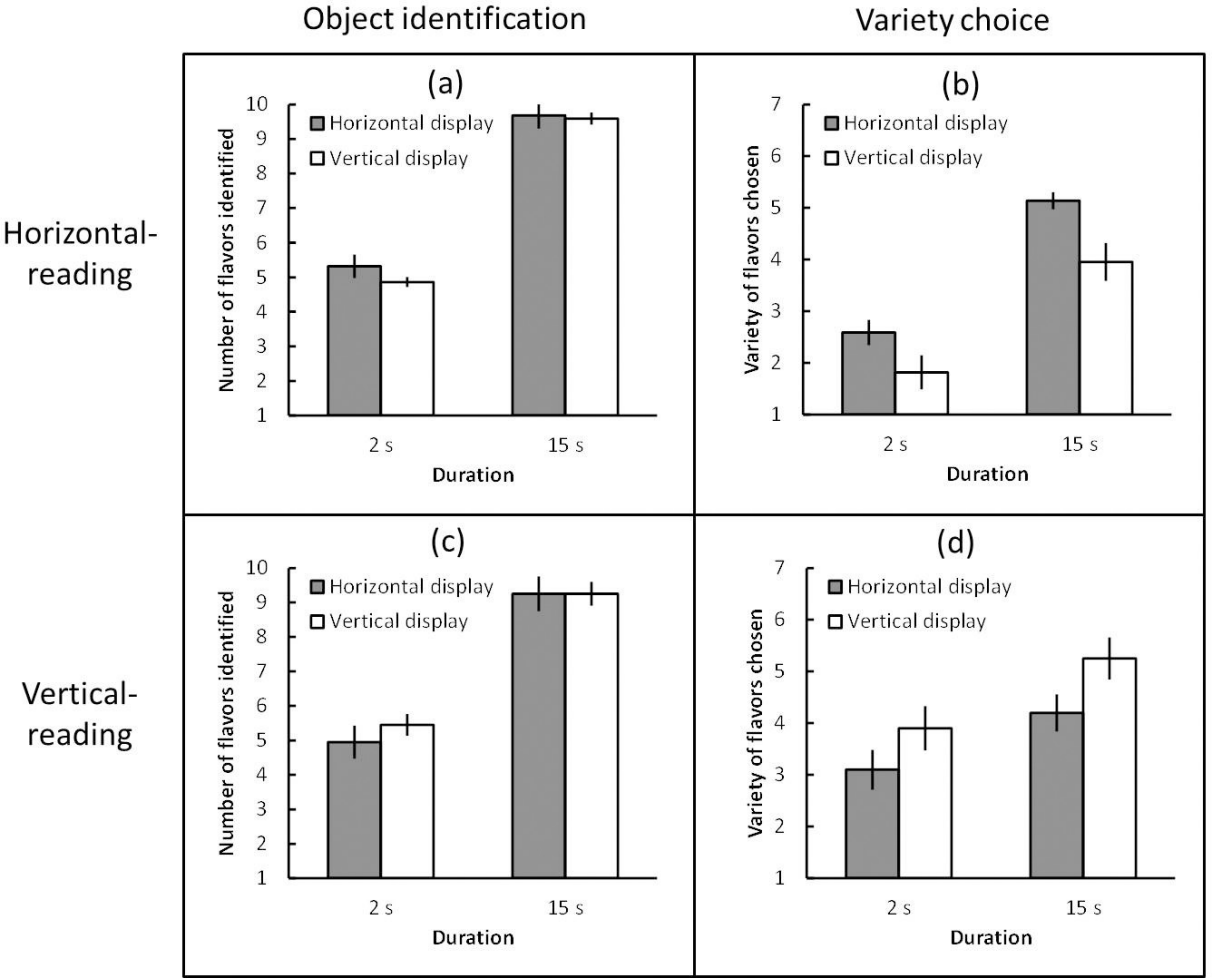
(a) Mean number of flavors identified (object identification) under the horizontal-reading condition, (b) mean variety of flavors chosen (variety choice) under the horizontal-reading condition, (c) mean object identification under the vertical-reading condition, and (d) mean variety choice under the vertical-reading condition in Experiment 2. Error bars indicate standard error of the mean.

The number of flavors that participants identified (object identification) and the variety of flavors that participants chose (variety choice) were averaged across participants for each condition. A mixed-design 2 (reading direction: horizontal and vertical) × 2 (display direction: horizontal and vertical) × 2 (duration: 2s and 15s) Bayesian ANOVA was first conducted to evaluate object identification (Fig 6A and 6C). The data provided evidence that all the interactions favored the null (*B*_*10*_ < 1.00, *partial η*^*2*^ < 0.16), but extreme evidence for a main effect of duration (*B*_*10*_ = 2.70 × 10^44^, *partial η*^*2*^ = 0.84); the other main effects favored the null (*B*_*10*_ < 1.00, *partial η*^*2*^ < 0.01).

For variety choice (Fig 6B and 6D), the three-way Bayesian ANOVA provided extreme evidence for the interaction between reading direction and display direction (*B*_*10*_ = 7.82 × 10^4^, *partial η*^*2*^ = 0.51), which was of most interest in this experiment. To explore the effect of display direction under each reading condition, a within-subject 2 (display direction: horizontal and vertical) × 2 (duration: 2 s and 15 s) Bayesian ANOVA was conducted. Under the horizontal-reading condition, the data provided moderate evidence for an effect of display direction (*B*_*10*_ = 6.65, *partial η*^*2*^ = 0.65), extreme evidence for an effect of duration (*B*_*10*_ = 5.70 × 10^11^, *partial η*^*2*^ = 0.79), and anecdotal evidence that the interaction between these factors favored the null (*B*_*10*_ = 1/2.40, *partial η*^*2*^ = 0.05). Under the vertical-reading condition, the data provided moderate evidence for an effect of display direction (*B*_*10*_ = 8.20, *partial η*^*2*^ = 0.41) and extreme evidence for an effect of duration (*B*_*10*_ = 18.80 × 10, *partial η*^*2*^ = 0.38), but moderate evidence that the interaction between these factors favored the null (*B*_*10*_ = 1/3.13, *partial η*^*2*^ = 0.02). Post-experimental interviews indicated that no participant had noticed the relationship between the reading and choice tasks prior to debriefing.

First, participants identified more flavors and chose a greater variety of flavors in the 15 s trial than in the 2 s trial under both the horizontal-reading and vertical-reading conditions. This suggested that the 15 s duration was sufficient for participants to identify nearly all varieties in the assortment. Second, and most interestingly, variety choice increased for both horizontal and vertical displays when participants were primed with the corresponding reading conditions. As the participants were exposed to horizontal and vertical displays in random order, the results do not simply reflect motor priming of eye movements.

These results suggest that horizontal and vertical reading influences the direction of visual processing, making subsequent product choice more efficient when the display is oriented in the corresponding direction. The display advantages observed in Experiment 2 provide strong evidence for the hypothesis that reading habits modulate the directional advantage of displays in terms of choice of varieties. At the same time, the immediately preceding reading experience can flexibly modulate the advantageous direction of assortment displays for Japanese readers.

In the next experiment, I explored the robustness of the findings of Experiment 2. As I manipulated independent variables among the participants of Experiment 2, an order effect might potentially have influenced the results. Although I sought to compensate for this by randomizing the conditional order among participants, I thought it useful to explore whether the same results would be obtained using a between-subjects design, as employed in a previous study [7].

## Experiment 3

### Method

I recruited 144 Japanese participants (69 males, mean age = 20.26 years, SD = 1.27 years) from the student community. All participants reported normal or corrected-to-normal visual acuity. All were blinded to the purpose of the study.

The stimuli, apparatus, and procedure were the same as those of Experiment 2, except that the display direction and duration differed among participants, of whom 80 were pseudo-randomly assigned to the horizontal-reading condition with the constraint that each display direction × duration interaction has the same sample size (i.e., 20 participants for each display direction × duration), and the other 64 to the vertical reading condition (i.e., 16 participants for each condition). All participants underwent only a single trial.

### Results and discussion

**Fig 7:**
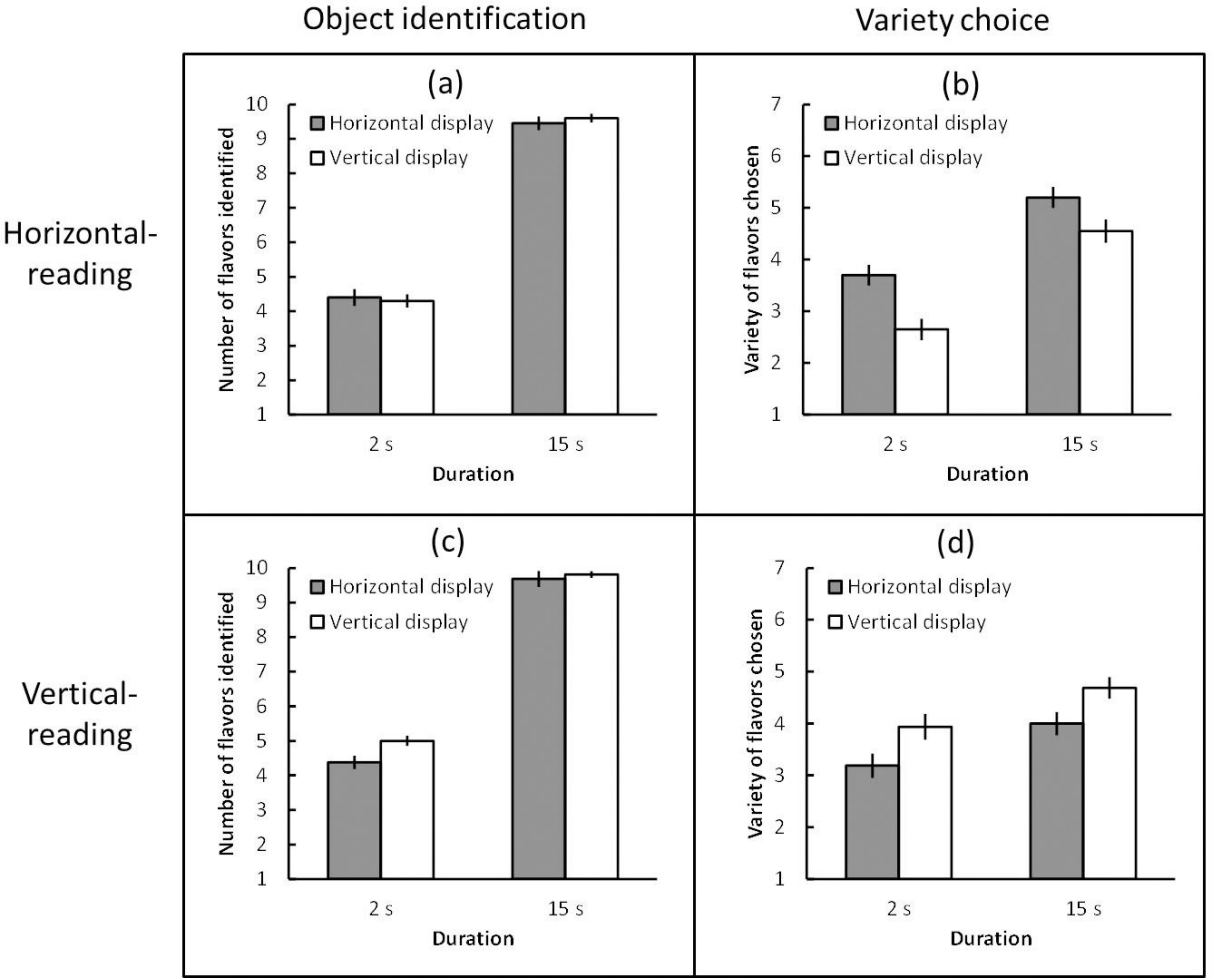
(a) Mean number of flavors identified (object identification) under the horizontal-reading condition, (b) mean variety of flavors chosen (variety choice) under the horizontal-reading condition, (c) mean object identification under the vertical-reading condition, and (d) mean variety choice under the vertical-reading condition in Experiment 3. Error bars indicate standard error of the mean.

The number of flavors that participants identified (object identification) and the variety of flavors chosen (variety choice) were averaged across participants for each condition. A between-subjects 2 (reading direction: horizontal and vertical) × 2 (display direction: horizontal and vertical) × 2 (duration: 2s and 15s) Bayesian ANOVA was first performed to explore object identification (Fig 7A and 7C). Because the data provided anecdotal evidence for the three-way interaction (*B*_*10*_ = 2.20, *partial η*^*2*^ = 0.03), a between-subjects 2 (display direction: horizontal and vertical) × 2 (duration: 2 s and 15 s) Bayesian ANOVA was performed under each reading condition. Under the horizontal-reading condition, the data provided moderate evidence that the effect of display direction favored the null (*B*_*10*_ = 1/4.31, *partial η*^*2*^ = 0.00), and extreme evidence of an effect of duration (*B*_*10*_ = 15.37 × 10^35^, *partial η*^*2*^ = 0.90). The data provided anecdotal evidence that the interaction between these factors favored the null (*B*_*10*_ = 1/2.81, *partial η*^*2*^ = 0.01). Under the vertical-reading condition, the data provided moderate evidence that the display direction favored the null (*B*_*10*_ = 1/3.42, *partial η*^*2*^ = 0.07), but extreme evidence of the effect of duration (*B*_*10*_ = 2.12 × 10^32^, *partial η*^*2*^ = 0.93). The data provided anecdotal evidence that the interaction between these factors favored the null (*B*_*10*_ = 1/1.47, *partial η*^*2*^ = 0.03).

In terms of variety choice (Fig 7B and 7D), the three-way Bayesian ANOVA indicated extreme evidence for the interaction between reading direction and display direction (*B*_*10*_ = 7.44 × 10^3^, *partial η*^*2*^ = 0.15), which was of most interest in this experiment. To explore the effect of display direction under each reading condition, a between-subjects 2 (display direction: horizontal and vertical) × 2 (duration: 2 s and 15 s) Bayesian ANOVA was performed under each reading condition. Under the horizontal-reading condition, the data provided moderate evidence of an effect of display direction (*B*_*10*_ = 9.11, *partial η*^*2*^ = 0.17) and extreme evidence of an effect of duration (*B*_*10*_ = 2.72 × 10^7^, *partial η*^*2*^ = 0.45), but anecdotal evidence that the interaction between these factors favored the null (*B*_*10*_ = 1/2.34, *partial η*^*2*^ = 0.01). Under the vertical-reading condition, the data provided moderate evidence of an effect of display direction (*B*_*10*_ = 6.58, *partial η*^*2*^ = 0.13) and strong evidence of an effect of duration (*B*_*10*_ = 12.60, *partial η*^*2*^ = 0.15), but anecdotal evidence that the interaction between these factors favored the null (*B*_*10*_ = 1/2.88, *partial η*^*2*^ = 0.00). Post-experimental interviews indicated that no-one noticed the relationship between the reading and choice tasks prior to debriefing.

Although the independent variables were manipulated among participants, as in the work of Deng and colleagues [7], our results exhibited similar patterns; both horizontal and vertical display advantages were apparent under equivalent reading conditions. Thus, the findings of Experiment 2 were robust; the horizontal display advantage is vulnerable to directional reading habits, especially prior reading experience. It appears that culture has effects on the horizontal display advantage, which has arisen by default from horizontally favored visual characteristics.

## General discussion

The present study investigated whether directional advantage of display orientation in terms of variety of product choice was susceptible to cultural difference in reading habits; I performed controlled laboratory experiments. First, in contrast to previous studies focusing on Westerners [7], the horizontal display advantage in terms of variety choice was not evident, even though the experiment adopted a very similar paradigm to that used in previous work (Experiments 1A and 1B). Second, for Japanese readers the advantageous direction of the display was flexibly modulated according to the immediately preceding visual and reading experience (Experiments 2 and 3). Specifically, the directional advantage of display orientation depended on whether participants had read horizontal or vertical text immediately prior to the choice task. Supporting my hypothesis, these results demonstrate that horizontal displays are not always advantageous; rather, cultural differences in reading habits modulate the relative advantages of horizontal and vertical displays in terms of product choice.

I would like to emphasize that my findings (no advantage afforded in Experiments 1A and 1B, but both horizontal and vertical advantages evident in Experiments 2 and 3) cannot be explained solely by the suggestion of Deng and colleagues [7] that visual characteristics favoring the horizontal direction explain the horizontal display advantage. My evidence does not refute this suggestion but, rather, advances the proposition that reading habits potentially modulate any horizontal display advantage. That is, the horizontal display advantage is not a unitary phenomenon mediated by a single factor. Culture affects the horizontal display advantage that has arisen inherently from horizontally favored perception. Although the present study demonstrated general cultural influences on product choice, future research should explore how culture plays a role in the assortment processing, or whether covert shifting of visual attention indeed contributes to the current findings. For clues about this issue, it is notable that the horizontal and vertical display advantages in Experiments 2 and 3 persisted for 2–15 s when it came to choosing flavors, even though participants identified flavors with equal efficiencies when presented in horizontal and vertical displays; also, the 15-s display duration allowed identification of almost all flavors. This finding suggests that reading habits may directly influence choice among varieties, rather than mediating identification of assortment components. Alternatively, the representations of the horizontal and vertical assortments may have qualitatively differed from one another, although they were quantitatively equivalent. Furthermore, future research on Westerners, who engage in only horizontal reading, may reinforce and embellish the claims presented here by indicating whether the horizontal display advantage can be modulated by short-term reading experiences (i.e., vertical reading immediately before the choice task, as in Experiments 2 and 3) or long-term reading experiences (i.e., horizontal reading).

When a horizontal display advantage was in play (under the horizontal-reading conditions of Experiments 2 and 3), the variety of flavors chosen from the horizontal display (2.59 and 5.14 for the 2-s and 15-s durations of Experiment 2; 3.70 and 5.20 for these durations in Experiment 3) did not increase compared to when the advantage was absent (3.90 and 5.60 in Experiment 1A; 3.76 and 5.57 in Experiment 1B). Rather, the variety of flavors chosen from the vertical display under the horizontal-reading conditions of Experiments 2 and 3 (1.82 and 3.95; 2.65 and 4.55, respectively) tended to be lower than those of Experiments 1A and 1B (3.90 and 3.58; 3.69 and 5.67, respectively). Similarly, when a vertical display advantage was in play (under the vertical-reading conditions of Experiments 2 and 3), the variety of flavors chosen from the vertical display (3.90 and 5.25 for the 2-s and 15-s durations of Experiment 2; 3.94 and 4.69 for these durations of Experiment 3) tended to be the same as when the advantage was absent (Experiments 1A and 1B). Also, the variety of flavors chosen from the horizontal display under the vertical-reading conditions of Experiments 2 and 3 (3.10 and 4.20; 3.19 and 4.00, respectively) tended to be less than those of Experiments 1A and 1B. Thus, the results consistently suggest that variety choices from both horizontal and vertical displays were suppressed when participants were primed with incongruent reading conditions in Experiments 2 and 3, although direct comparisons among experiments may be inappropriate because of methodological differences. In this context, the horizontal display advantage may reflect a form of suppression of variety choice in terms of the vertical display, rather than facilitation of the horizontal display. Given that participants in Experiments 1A and 1B might already be primed to read in both horizontal and vertical directions according to their daily habits, variety choice for both displays would moderately indicate this advantage, although no difference was detected between display directions. When participants in Experiments 2 and 3 activated a single reading direction, suppression (or a disfluent effect) of variety choice became predominant for the incongruent direction, as compared to facilitation (or a fluent effect) for the congruent direction, thus making the difference between display directions salient. Therefore, even if this was the case, current evidence does not conflict with the claim that reading culture modulates the horizontal display advantage. More work is needed.

Previous research has suggested that culture, which comprises the sum of the knowledge, beliefs, morals, customs, and other capabilities and habits acquired by the members of a society, has important effects on consumer behavior [26,27]. For example, preceding studies have reported that culture can influence emotion or motivation. Consumers’ attitudes toward foreign products, advertising, and preferred sources of information differ among cultures based on values and norms [28–30], leading to different patterns of consumption. Furthermore, people who belong to collectivistic cultures tend to value uniformity in their behaviors or attitudes when consuming, while people from individualistic cultures tend to value diversity [26,27]. The present study demonstrated the reliable influence of culture (reading habits) in the context of assortment processing.

## Conclusion

The supposed advantage imparted by directionally organized assortment is susceptible to cultural differences in consumer reading habits. In particular, for Japanese readers the variety of flavors chosen was enhanced when the direction of the display matched the direction of text read immediately prior to the choice task. It is known that in-store visual factors, such as shelf space [31,32], number and position of shelf facings [33,34] and horizontal displays [7] can influence consumer decision-making. My study not only contributes to this existing theoretical framework but also reveals a novel relationship between display direction and cultural factors that influences how consumers choose from product assortments. This finding has practical implications. For example, it may be important to maintain a consistent direction between the external packaging design and the internal allocation of products, or between visual labels on a shelf and the allocation of products in the shelf. According to de Mooij and Hofstede [35], retailing strategies for one country cannot be extended to other countries without adaptation to account for cultural differences in consumer behavior. Based on the current findings, retailers or marketers should aim to match the directional design in retail settings to the consumers’ reading culture.
